# Successful Treatment of Microsporidial Keratoconjunctivitis (MKC) With a Combination of Topical Voriconazole 1% and Gatifloxacin 0.5%: A Large Case Series of 29 Patients

**DOI:** 10.7759/cureus.49247

**Published:** 2023-11-22

**Authors:** Balamurugan Ramatchandirane, Mohan Kumar A, Yamini Marimuthu, Deepthi Slesser Nicodemus, Mano Chandrika Yarra

**Affiliations:** 1 Ophthalmology, All India Institute of Medical Sciences (AIIMS), Mangalagiri, IND; 2 Medical Microbiology, All India Institute of Medical Sciences (AIIMS), Mangalagiri, IND; 3 Community and Family Medicine, All India Institute of Medical Sciences (AIIMS), Mangalagiri, IND

**Keywords:** mixing and mashing technique, combination of voriconazole and gatifloxacin, mkc, microsporidial keratoconjunctivitis, empirical treatment, scraping technique, gatifloxacin, voriconazole, keratitis, microsporidiosis

## Abstract

Objective: This study aims to report the successful treatment of microsporidial keratoconjunctivitis (MKC) with the combination of topical drops of voriconazole (1%) and gatifloxacin (0.5%) in all 29 patients. Demography, clinical profile, and previous treatment history were also analyzed.

Methods: A retrospective, non-comparative case series of all Gram stain-proven MKC from September 2021 to October 2022 was included in this study. Patients were given antimicrobials such as topical drops of voriconazole 1%, gatifloxacin 0.5%, or a combination of both in 29 patients based on the treatment response. Topical steroids were added to 31 patients for corneal haziness.

Results: A total of 33 patients were found to be positive for microsporidiosis confirmed by Gram staining. Twenty-four (72.7%) were men and nine (27.3%) were women. The mean age was 34.45±12. The presenting symptoms were mainly redness in 30 patients (90.9%), followed by watering in 13 (39.4%), foreign body sensation in 10 (30.3%), etc. Among the 23 patients (69%), a history of risk factors was identified, with 17 patients (51.5%) specifically reporting dust exposure as a major cause. MKCs were successfully treated with antimicrobials such as voriconazole 1% in three patients, gatifloxacin 0.5% in one patient, and a combination of both in 29 patients. Topical steroids were added to 31 patients for corneal haziness. At the last follow-up, a visually insignificant nummular corneal scar was noted in six patients. No drop in vision was noted in any of these patients at the end of the follow-up. No cases progressed to stromal keratitis and no surgical intervention was required in any cases.

Conclusions: We successfully treated all 29 cases with a combination of voriconazole and gatifloxacin without requiring any surgical intervention or encountering stromal complications. This successful treatment in all 29 cases offers valuable insights into the potential of this drug combination, possibly attributable to its additive action or broad-spectrum coverage across various species.

## Introduction

Microsporidial keratoconjunctivitis (MKC) is a rare infectious entity infecting the cornea and conjunctiva caused by *Microsporidium* species. Microsporidia are unicellular, spore-forming, obligate intracellular parasites, initially classified as protozoa but later it is now reclassified as fungi based on genomic evidence [1]. There are more than 1,000 species identified under different genera of microsporidia but only a few genera like *Vittaforma*, *Nosema*, *Anncaliia*, *Enterocytozoon*, *Pleistophora*, *Trachipleistophora*, and *Encephalitozoon* are frequently involved in infections of the eye, muscles, intestine, central nervous system, and kidneys [2]. Eye infection with microsporidium was first reported in 1973 by Ashton and Wirasinha [3] followed by many case reports and case series. Microsporidium causes infections in various parts of the eye including epithelial keratoconjunctivitis, stromal keratitis, and scleritis [4]. Many drugs are described in the literature for the treatment of MKC, but no standard treatment protocol is mentioned in the literature [4]. In this study, the authors have described the successful management of MKC in 33 patients with the combination of voriconazole and gatifloxacin in 29 patients, voriconazole only in three patients, and gatifloxacin only in one patient, along with topical steroids without any progression to stromal or other ocular-related complications. In this paper, the authors have also described the demography, clinical profile, previous medical drugs, and treatment outcome of MKC in a tertiary-care center in South India.

## Materials and methods

**Figure 1:**
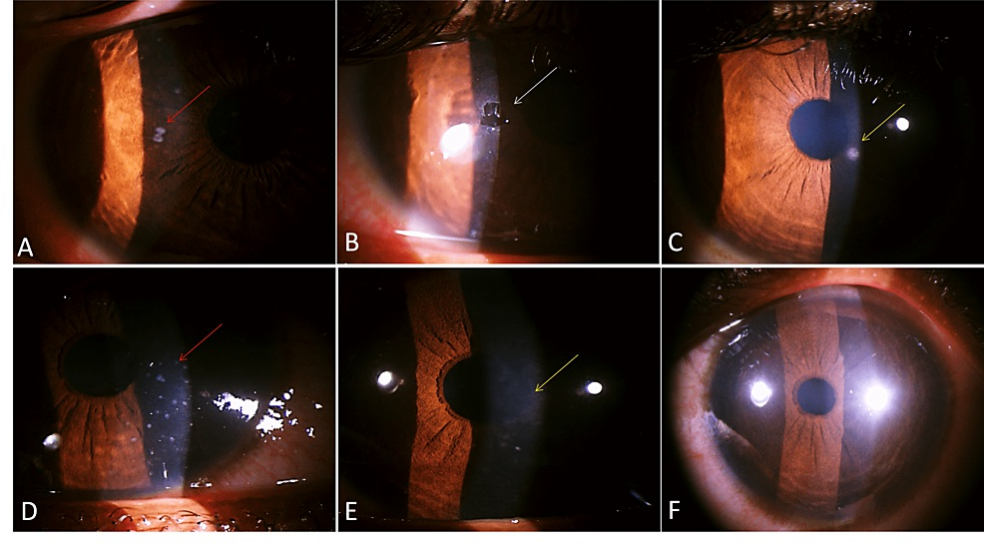
(A, D) Multiple, raised, greyish-white lesions (red arrow) suggestive of SPKs on the corneal epithelium. (B) Epithelial defect (white arrow) after diagnostic corneal scraping. (C) Healed lesions with corneal scar (orange arrow) at the last follow-up. (E) Healing lesions with corneal haziness during the course of treatment (orange arrow). (F) Complete clear cornea after adding topical steroid along with antimicrobials

**Figure 2:**
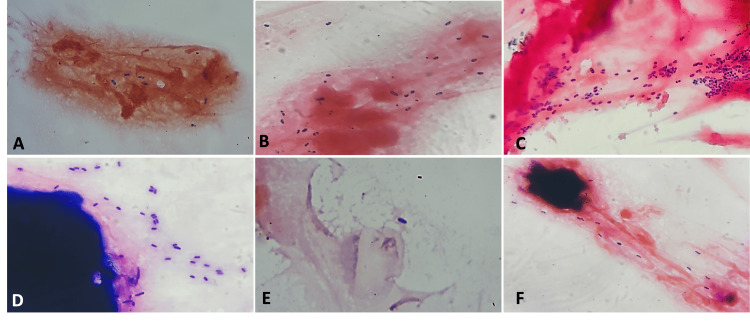
(A-F) Light microscopy images of oval-shaped microsporidial spores by Gram staining of six different patients which were taken under camera with various magnification

The clinical profile, demography, and treatment outcome of the patients with laboratory-confirmed MKC were reviewed retrospectively for one year from September 2021 to October 2022 in a tertiary-care center in South India. The study was approved by the Institutional Ethics Committee of All India Institute of Medical Sciences, Mangalagiri (approval number: IEC/2022-23/247, approval date: 20.1.2023). Patients with clinical features suspicious of microsporidiosis such as multiple, elevated, greyish, coarse superficial punctate lesions (SPKs) (Figure 1A, 1D) underwent corneal scraping. For scraping the lesions, we used a sterile 26-gauge needle which was bent to 45 degrees toward the bevel side using its own cap. Topical anesthetic drops (proparacaine 0.5%) were instilled, and under slit lamp magnification, the most prominent lesions were chosen for scraping. Using the needle tip with the bevel facing down, the lesions along with the little surrounding epithelium were scraped (Figure 1B) by using repeated up-and-down strokes of the needle (Video 1). Precautions should be taken while scraping as it should involve only the epithelium and should not be deeper and penetrate the stroma. Scraping a small amount of surrounding epithelium mixed with the lesions will provide an adequate sample volume. The scraped material was then mashed and ground on the glass slides to make the circular smear (mixing and mashing technique) (Video 1). The specimen slide was then sent to the microbiology laboratory for diagnostic confirmation. The labeled slides were stained with Gram stain to observe oval spores morphologically similar to microsporidium under the light microscope (Figure 2A-2F). Upon microbiological confirmation on the same day, patients were started on topical antimicrobials, namely, voriconazole 1%, gatifloxacin 0.5% every one hour on the same day and frequency decreased to six times or four times based on the response on the next day, and topical steroids with either fluorometholone 0.1% or prednisolone 1% four to six times a day were started based on the corneal haziness on next day, i.e., day 1 after ensuring that the epithelial defect due to corneal scraping was healed. Topical antimicrobials and steroids were tapered down until the complete disappearance of SPKs and corneal haze (Figure 1E-1F). Patients were asked to follow up on day 1, day 3 or 5, or day 7 based on the response and weekly after that till the resolution of the SPKs. Treatment was considered successful when there was complete resolution of the SPKs with no active inflammation.

**Video 1:** Mixing and mashing technique for corneal scraping for SPKs

Statistical analysis

The data collected was entered in Microsoft Excel (Microsoft Corporation, Washington, USA) and analyzed using STATA statistical software version 14 (StataCorp LCC, Lakeway Drive College Station, Texas, USA). Categorical variables were summarized as frequencies and proportions, whereas continuous variables were summarized as mean (SD) or median (IQR) based on the distribution of data.

## Results

**Figure 3:**
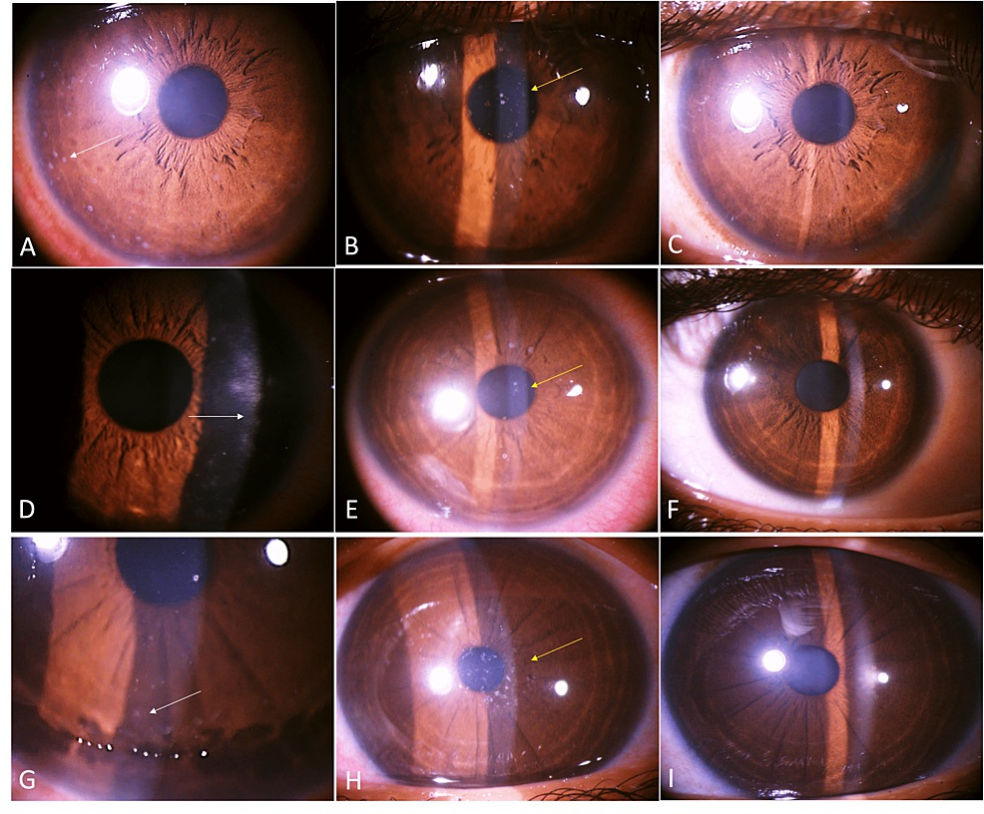
(A, D, G) SPKs in the peripheral of the cornea at the initial presentation (white arrow). (B, E, H) Cluster of new SPKs in the center of the cornea (yellow arrow) after disappearance from the periphery of the cornea. (C, F, I) Complete clearance of the central lesions at the last follow-up

**Figure 4:**
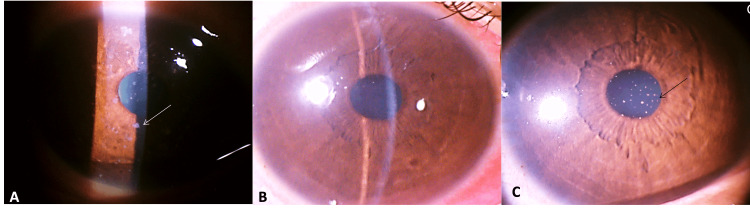
(A) SPKs in the initial presentation (white arrow). (B) Lesions disappeared with antimicrobials on day 3 post treatment. (C) The appearance of KPs (black arrow) after resolution of the SPKs on day 5 post treatment

A total of 33 clinically suspected MKC with multiple elevated, greyish, coarse SPKs (Figures 1A, 1D, 3A, 3D, 3G, 4A) with Gram staining-proven microsporidial spores (Figure 2A-2F) in corneal scrapings were included in the study. Among them, there were 24 (72.7%) men and nine (27.3%) women. The mean±SD age of the study participants was 34.45±12.0. The right eye was involved in 14 (42.4%) and the left eye was involved in 19 (57.6%). No bilateral eye involvement was noted. The symptoms with which the patients presented were redness in 30 (90.9%), watering in 13 (39.4%), foreign body sensation in 10 (30.3%), pain in eight (24.2%), decreased vision in three (9.1%), discharge in two (6.1%), and itching in one (3%). History of ocular exposure to dust was present in 17 (51.5%) patients, rainwater/dirty water was present in five (15.1%), and insects in one (3.0%) of the patients. A history of exposure to dust or any other similar risk factors was not present in 10 (30.3%). The mean±SD intraocular pressure (IOP) measured by non-contact tonometry (NCT) at presentation was 16.2±3 mmHg, and it ranged from 12 mmHg to 25 mmHg. Conjunctival follicles were present in six (18.2%) patients. Migration of the lesions (SPKs) toward the center from the periphery of the cornea after peripheral clearing of the lesion was seen in eight patients (Figure 3B, 3E, 3H). Keratic precipitates (KPs) were present in two (6%) patients (Figure 4C). Pre-auricular lymphadenopathy was present in four (12.1%) patients. Six (18.2%) patients had type II diabetes mellitus among which four had uncontrolled diabetes. Patients had a previous history of treatment with topical antibiotics such as moxifloxacin in 10 (30.3%), ofloxacin in three (9%), ciprofloxacin in two (6%), gatifloxacin in one (3%), tobramycin in five (15%), gentamicin in one (3%), chloramphenicol in one (3%), antivirals like acyclovir ointment in two (6%), ganciclovir ointment in three (9.1%), tablet acyclovir in two (6%), artificial tears in nine (27.3%), topical steroid in 13 (39.4%), and olopatadine in one (3%). After microbiological confirmation of microsporidial spores on Gram staining (Figure 2A-2F), the patients were treated with various antimicrobials such as voriconazole (1%) and gatifloxacin (0.5%), along with topical steroids with fluorometholone 0.1% or prednisolone 1%. Among 33 patients, topical voriconazole 1% alone was given to three (9.09%) patients; topical gatifloxacin 0.5% alone was continued in one (3.03%) who was already on gatifloxacin on presentation with a healing corneal scar; combined voriconazole 1% and gatifloxacin 0.5% was given in 29 (87.9%). Topical steroids were given to 31 (93%) patients based on corneal haziness (Figure 1E), topical prednisolone 1% was given to 11 (33.3%) patients in cases of significant haze, and fluorometholone 0.1% was given to 20 (60.6%) patients in cases of mild corneal haze. At the end of the last follow-up, the nummular scar was noted only in six (18.7%) patients (Figure 1C). The mean±SD IOP by NCT at the last follow-up was 15.7±2.7 mmHg and it ranged from 12 mmHg to 21 mmHg. At the last follow-up, 31 patients achieved the best corrected visual acuity (BCVA) of 20/20 in the Snellen chart, one patient achieved the pre-illness documented vision of 20/40 (BCVA), and one patient was lost to follow-up for performing BCVA. No patient has lost a single line with respect to the documented baseline vision. The mean time taken for the early signs of treatment response in terms of SPKs like shrinking in size or decreasing in number or disappearance of SPKs was 1.25±0.43. The median (IQR) time taken for complete resolution after starting medications was four (three to six) days ranging from one day to 10 days.

**Table 1:** Demography and clinical profile of MKC patients OD: right eye, OS: left eye, SPKs: superficial punctate lesions, KPs: keratic precipitates, MKC: microsporidial keratoconjunctivitis

Variable		Frequency (percentages) N=33
Sex	Male	24 (72.7)
	Female	9 (27.3)
Eye	OD	14 (42.4)
	OS	19 (57.6)
Presence of symptoms	Redness	30 (90.9)
	Pain	8 (24.2)
	Foreign body sensation	10 (30.3)
	Decreased vision	3 (9.1)
	Watering	13 (39.4)
	Discharge	2 (6.1)
	Itching	1 (3.0)
H/o foreign body exposure	Rainwater/dirty water	5 (15.1)
	Dust	17 (51.5)
	Insect	1 (3.0)
	No history	10 (30.3)
Initial presentation of SPKs	Central	19 (57.6)
	Peripheral	13 (39.4)
	Diffuse	1 (3)
Presence of follicles	Yes	6 (18.2)
Presence of KPs	Yes	2 (6.0)
Presence of corneal haze during the course	Yes	18 (54.5)
Presence of pre-auricular lymph node	Yes	4 (12.1)
Type II diabetes mellitus	Present	6 (18.2)
Treatment history	Moxifloxacin	10 (30.3)
	Gatifloxacin	1 (3.0)
	Tobramycin	5 (15.1)
	Gentamicin	1 (3.0)
	Ciprofloxacin	2 (6.1)
	Ofloxacin	3 (9.1)
	Chloramphenicol	1 (3.0)
	Acyclovir 3% ointment	2 (6.1)
	Oral acyclovir	2 (6.1)
	Ganciclovir	3 (9.1)
	Artificial tears	9 (27.3)
	Topical steroids	13 (39.4)
	Miscellaneous (olopatadine)	1 (3.1)
Treatment after diagnosis	Voriconazole 1% alone	3 (9.09)
	Gatifloxacin 0.5% alone	1 (3.03)
	Voriconazole + gatifloxacin	29 (87.9)
	Prednisolone 1%	11 (33.3)
	Fluorometholone 0.1%	20 (60.6)
Corneal scar at last follow-up	Present (nummular scar)	6 (18.7)

## Discussion

MKC is generally considered a rare entity and is very often misdiagnosed and treated as viral conjunctivitis [5]. Its distributions are relatively higher in some geographical areas like South India as was evident from more literature published in the southern states of India [2,6-10]. The diagnosis entirely depends upon the microbiological confirmation of the characteristic oval-shaped microsporidial spores seen in Giemsa staining (sensitivity of 73.3%), Gram staining (sensitivity of 90%), modified acid-fast staining (sensitivity of 93.3%), and KOH + CFW staining (sensitivity of 96.7%) [11]. In the absence of KOH + CFW staining, we used only Gram staining for confirmation, which gives reasonably high positivity. The patients usually present with typical corneal lesions such as coarse, greyish-white, raised SPKs in the cornea along with conjunctival follicles and pre-auricular lymphadenopathy in a few cases.

We have seen a strange pattern of migrating lesions in eight patients from the peripheral to the central cornea during the course of treatment, while the peripheral cornea cleared off completely from the lesions (Figure 3B, 3E, 3H). This centripetal migration or distribution has occurred possibly because of the centripetal migration of the normal epithelium from the limbus to the central cornea as described in the XYZ hypothesis of corneal maintenance proposed by Thoft et al. [12]. This kind of centripetal distribution or migration of the lesions to the central cornea is more desquamatory in appearance and has also caused deterioration of vision after initial relief of the symptoms. Later, with the same antimicrobials and frequent or stronger steroids, these central lesions are resolved without any significant corneal haze (Figure 3C, 3F, 3I), and hence reassurance to the patient is necessary.

There is currently no standard treatment protocol available for microsporidial keratitis, and there are various studies [5-10,13] showing that MKC responds to fluoroquinolones (moxifloxacin, gatifloxacin, etc.), antifungals such as voriconazole, fluconazole, and itraconazole, anti-parasitic drugs such as oral albendazole and fumagillin, and antiseptics such as polyhexamethylene biguanide and dibrompropamidine isethionate. In our scenario, the treating clinician who has migrated from one region of India to another region and joined this newly established hospital has successfully treated MKC with voriconazole 1% drops alone in the initial three patients. Antifungal eyedrops like voriconazole have been reported to cure MKC [14,15], but we also experienced two other cases of MKCs that did not respond to voriconazole 1% alone in terms of increasing SPKs, limbitis, diffuse conjunctival chemosis, and eyelid swelling which all responded promptly after adding gatifloxacin. Therefore, we thought of the possibility that some species are likely resistant to voriconazole and some species are likely sensitive to gatifloxacin. Owing to the nature of some species with varying sensitivity to voriconazole, we have added gatifloxacin along with voriconazole, and we treated MKC successfully in the next 27 cases.

Among these 29 cases, one patient was prescribed combined voriconazole and gatifloxacin, but the patient was started with gatifloxacin only for two days because of the unavailability of voriconazole. The symptoms worsened with increased SPKs and hence voriconazole was insisted on to start with, and the SPKs resolved completely in two days. This shows some microsporidial species are likely resistant to gatifloxacin and sensitive to voriconazole. In one case, the patient was already started on acyclovir and gatifloxacin from an outside hospital with healing lesions with significant haziness in the periphery of the cornea. Thus, because of healing lesions, we have continued gatifloxacin 0.5% alone without adding voriconazole, stopped acyclovir, and added topical prednisolone 1% for the corneal haziness, with which the SPKs resolved completely without any corneal haziness in nine days. This shows that gatifloxacin alone is likely the treatment for some microsporidial species. Hence, we believe that a combination regimen of voriconazole and gatifloxacin could be successful in MKC attributable to its broader species coverage and also due to its additive effect. A patient showed resolving SPKs with a combined topical voriconazole plus gatifloxacin treatment. However, the patient sought a second opinion at an outside hospital and underwent therapeutic epithelial debridement. This patient has returned to us because of intolerable pain due to an epithelial defect caused by epithelial debridement and has followed up with us thereafter. The patient was restarted with topical voriconazole and gatifloxacin but stopped topical steroids for two weeks because of an epithelial defect, and the patient developed mild corneal haziness with a BCVA of 20/20 at the last follow-up. This same patient has recurred with similar complaints with SPKs after two months and was treated successfully with combined voriconazole 1% and gatifloxacin 0.5% with fluorometholone 0.1% four times a day in two weeks with a BCVA of 6/6 with a mild nummular corneal scar at the final follow-up. This was the only case of corneal recurrence noted in our study.

Many authors have described how mechanical debridement of the lesion could be effective with or without other antimicrobials [1,4,14,16,17]. We have seen the resolution of SPKs in all patients with medical management only. Therapeutic epithelial debridement is a painful invasive procedure and healing takes a relatively longer duration and also poses the risk of deeper penetration of infection and secondary infections [14]. Depending upon the severity of corneal haziness during the course of the treatment, patients were started with either a lower-potential fluorometholone 0.1% four to six times a day or a higher-potential topical steroid with prednisolone 1% four to six times a day or shifting from fluorometholone to prednisolone in cases of inadequate response. The topical steroid was given to 31 patients because of corneal haziness on day 1, and we avoided topical steroid on the same day because of the epithelial defect made by the corneal scraping. In three patients, topical steroids were shifted from lower-potential fluorometholone 0.1% to higher-potential prednisolone 1% because of significant corneal haze. There was no exaggeration of the symptoms and signs after starting topical steroids along with antimicrobials.

Chan et al. [5] in their retrospective case series of six patients with MKC who were treated with antimicrobials without any topical steroids developed sub-epithelial scars in all patients. They have also experienced that topical steroids without any antimicrobials lead to persistent microsporidial infections. We have used topical steroids for the presence of corneal haziness caused by inflammation in 31 patients, and a visually insignificant nummular scar was noted in six patients only. Two patients developed KPs (Figure 4C) after the resolution of the SPKs (Figure 4B), which subsequently disappeared with frequent topical steroids. The appearance of KPs after complete resolution of SPKs could be immune-mediated as postulated by Loh et al. [13] and needs frequent topical steroids. Fifteen patients who were already on fluoroquinolones (10 moxifloxacin, two ciprofloxacin, and three ofloxacin) presented to us because of progressive symptoms with active SPKs, which all responded with combined voriconazole and gatifloxacin; hence, the role of other fluoroquinolones in the management of the microsporidium is not obvious. According to Loh et al. [13] in their retrospective studies of 134 eyes, resolution of lesions was seen in 99% of cases with the fluoroquinolones class of antibiotics, but they did not mention drug-wise among the fluoroquinolones. The prophylactic antibiotics after corneal scraping could be substituted with gatifloxacin in place of other antibacterial drops further highlighting its added role in this condition. The self-limiting nature of the disease has been described in much literature [8-10], but in our study, we noted that 23 patients came to us for a second opinion with deteriorating symptoms, despite already receiving treatment with medications from outside hospitals for some time. MKC could be a self-limiting disease, but it could have a long course [18], and treatment response depends upon the species and immunity of the patient [19]. The mean time taken for the early sign of treatment response in terms of SPKs like shrinking in size or decreasing in number was 1.25±0.43 days in our study. Hence, we recommend timely medical intervention with antimicrobials to reduce the patient's suffering. A corneal defect after diagnostic corneal scraping was re-epithelialized in all patients presented on day 1. The median time taken for complete resolution of the lesions was four (three to six) days in the range of 1-10 days which is lesser when compared to the time taken for resolution as mentioned in other literature as seven to 60 days by Khurana et al. [4] and 2-18 days by Das et al. [8]. We have noted that no cases have progressed to stromal keratitis or developed limbitis with combination medications, and no cases required surgical intervention for any other complications.

The main limitation of our study was the lack of a control group because of the retrospective, non-comparative design. Though we have successfully managed MKC with the combination of voriconazole and gatifloxacin in all 29 patients without any surgical intervention or major complications like stromal keratitis, a randomized comparative study is advocated to determine its effectiveness along with species identification. In addition, immune status was not assessed in the patients, and all patients were systemically well and have been presumed immunocompetent. Species determination was also not done due to a lack of diagnostic infrastructure.

## Conclusions

MKC is a rare infection affecting the cornea and conjunctiva but relatively common in some geographic regions, like South India, and it may be misdiagnosed as viral keratoconjunctivitis; hence, microbiological confirmation is mandatory for all SPKs for early treatment. We have found that dust falling in the eye is a major risk factor, as it was seen in 51% of the patients. Since there is no established protocol for the treatment of MKC, we have used the combination of voriconazole and gatifloxacin, which was successful in all 29 patients without any surgical intervention or any significant complications. This successful treatment offers valuable insights into the potential of this drug combination, possibly attributable to its additive action or broad-spectrum coverage across species.
